# Heterogeneous effects on body mass index in the “checkup championship”: A behavioral science-based health promotion program by health interest level

**DOI:** 10.1016/j.pmedr.2025.103153

**Published:** 2025-06-28

**Authors:** Takuya Yamada, Kumi Sugimoto, Hanae Nagata, Yoshiharu Fukuda, Koryu Sato, Naoki Kondo

**Affiliations:** a Teikyo University Graduate School of Public Health, 2-11-1, Kaga, Itabashi-ku, Tokyo, Japan, 173-8605 Tokyo, Japan; b Department of Social Epidemiology, Graduate School of Medicine and School of Public Health, Kyoto University, Kyoto, Japan; c Faculty of Policy Management, Keio University, Kanagawa, Japan

**Keywords:** Behavioral science, Nudge, Health education, Weight loss, Health concerns, Health disparities, Occupational health

## Abstract

**Objective:**

Behavioral science principles, including approaches such as gamification, commitment strategies, and nudges, are widely used in health promotion programs to prevent non-communicable diseases. These approaches are expected to influence behavior change regardless of health interest; however, their effectiveness remains unclear. This study evaluated the impact of a behavioral science-based health promotion program on body mass index (BMI) reduction across different levels of health interest.

**Methods:**

This study evaluated the “Checkup Championship,” a program that applies various behavioral science strategies to improve health checkup results for employees at Hakuhodo DY Group in Japan. Participants in the program in 2020 were compared with non-participants. Health interest was classified as low, middle, or high based on a single-question assessment. A linear regression model analyzed BMI changes between 2019 and 2020, using the inverse probability weighting of propensity scores to adjust for background differences between groups.

**Results:**

A total of 410 participants and 390 non-participants were included in the study. BMI reduction was greater among participants than non-participants (−0.36 kg/m^2^ vs. −0.12 kg/m^2^). A significant BMI reduction was observed in the middle (average treatment effect [ATE]: −0.30 kg/m^2^, 95 % confidence interval [CI]: −0.55, −0.06) and low health interest groups (ATE: −0.34 kg/m^2^, 95 % CI: −0.61, −0.08); however, no clear BMI reduction was seen in the high health interest group.

**Conclusions:**

The “Checkup Championship” demonstrated effectiveness, particularly among individuals with a lower health interest. Health programs incorporating diverse behavioral science strategies may help reduce health disparities.

## Highlights

• Behavioral science program lowers body mass index in low- and mid-health interest groups.
• No clear body mass index reduction observed in the high-health interest group.
• The study compares participants and non-participants in a workplace health program.
• A linear regression model analyzed body mass index changes from 2019 to 2020.
• The Behavioral science-based health programs can reduce health disparities.

## 1 Introduction

Non-communicable diseases among workers represent a global health problem (World Health Organization and Burton, 2010). Obesity, in particular, contributes to a higher incidence of diabetes, cardiovascular disease, cancer (Safaei et al., 2021), and increased mortality risk (Flegal et al., 2013; Global BMI Mortality Collaboration, 2016). To support weight control, collaboration among various sectors is recommended to promote healthy lifestyles (World Health Organization, 2023). Many countries have established guidelines for weight control (Semlitsch et al., 2019). Programs focused on diet and exercise for weight loss (Johns et al., 2014; Johnston et al., 2014; Joseph et al., 2019; Ma et al., 2017) and weight gain prevention (Peirson et al., 2014) have shown some effectiveness.

However, the effectiveness of healthy lifestyle practices and weight control programs depends on the characteristics of the target population. Individuals with lower health consciousness and motivation often show reluctance toward accessing health information (Iversen and Kraft, 2006) and are less likely to adopt health behaviors such as regular exercise (Iversen and Kraft, 2006; Pu et al., 2020) and fruit and vegetable consumption (Iversen and Kraft, 2006) in daily life (Nishizawa et al., 2024). Furthermore, lower subjective health scores have been reported in this population (Xue et al., 2020). Socioeconomic factors influence health interest (Nishizawa et al., 2024), with lower socioeconomic status (SES) associated with reduced motivation for weight control (Siu et al., 2011), infrequent weight management efforts (Wardle and Griffith, 2001), and lower perceived program effectiveness (Beauchamp et al., 2014). Therefore, expanding the development and dissemination of effective health programs targeting individuals with low health consciousness and motivation remains essential for addressing health disparities.

In the development and dissemination of health programs, integrating behavioral science theories and techniques is recommended (Glanz and Bishop, 2010; Bach Habersaat and Altieri, 2023). Approaches such as gamification, commitment strategies, incentivization, and insights from behavioral economics (Thaler and Sunstein, 2008) have recently been applied to health program development. Programs applying these diverse behavioral science principles have been implemented and evaluated for effectiveness in weight control (Kurtzman et al., 2018; Patel et al., 2021; Nagata et al., 2022; Takebayashi et al., 2022), smoking cessation (Littman et al., 2022; Nurchis et al., 2023), physical activity promotion (Agarwal et al., 2021; Boonmanunt et al., 2023; Takebayashi et al., 2024), and dietary behavior and food selection guidance (Arno and Thomas, 2016; Boonmanunt et al., 2023; Fergus et al., 2023; Gibson et al., 2024; Harbers et al., 2020; Nagatomo et al., 2019; Vermeer et al., 2014).

The “Checkup Championship” (Hakuhodo Inc, 2025), examined in this study, represents a workplace health promotion program in Japan. Designed as a workplace-based health promotion initiative, this program sets the health checkup day as a goal date and begins approximately one and a half months in advance. The purpose is to encourage participants to improve their health checkup results compared to the previous year (Hakuhodo Inc, 2025; Nagata et al., 2022). This program incorporates a range of behavioral science principles, including “ease of initiation” (related to choice architecture), “commitment,” “gamification,” “use of social norms,” and “timely program implementation,” drawing from the Easy, Attractive, Social, and Timely framework (The Behavioural Insights Team, 2024). Participation in the “Checkup Championship” has been associated with significant improvements in weight, body mass index (BMI), and abdominal circumference, with particularly notable benefits for individuals eligible for health guidance (Nagata et al., 2022).

These behavioral science approaches are expected to promote health behaviors and ultimately reduce health disparities, regardless of SES or health interest level (Roberto and Kawachi, 2015). Although previous studies have shown that programs applying nudge theory are effective for individuals and populations with low SES and contribute to reducing health disparities, others have reported no effect on reducing health disparities (Harbers et al., 2020; Schüz et al., 2021). Furthermore, the effectiveness of these behavioral science interventions for individuals with low health interests remains unclear.

This study aimed to evaluate the impact of participation in the “Checkup Championship” on weight loss based on varying levels of health interest. Because the “Checkup Championship” applies multiple behavioral science strategies, a weight loss effect is expected across all health interest levels. However, differences in effectiveness based on health interests have not been verified.

## 2 Methods

### 2.1 Participants and recruitment

The study targeted 3172 employees of the Hakuhodo DY Group (Hakuhodo DY Holdings, Hakuhodo, and Hakuhodo DY Media Partners) with available health checkup data from both 2019 and 2020. Recruitment began one and a half months before the 2020 regular health checkups. Invitations were sent via e-mail, and posters were displayed within the company. Employees could register through the company intranet system before their scheduled health checkup. Eligibility criteria included all employees with 2019 health checkup data, regardless of position, age, or employment status. The participation fee was free of charge.

### 2.2 Intervention: Program components of the “Checkup Championship”

The intervention group included employees who participated in the 2020 “Checkup Championship” program, held from July 21 to September 29, 2020. This workplace health promotion program was based on multiple behavioral science principles, primarily incorporating elements of gamification, commitment strategies, and incentivization, rather than relying solely on nudge theory. Weekly e-mails were sent to all employees, with the content varying each week. Messages included encouragement to register (leveraging timeliness and ease of participation), information on metabolic syndrome and lifestyle improvements (providing knowledge), examples of employees engaging in healthy behaviors (social norm/modeling), health declarations (commitment element), and insights from program planners.

Participation required employees to declare their involvement via the company's intranet, serving as a soft commitment technique. Individual results were provided online to participants 3 months after the health checkups. Feedback included radar charts with enhanced visual elements highlighting improvement rates across various health indicators, serving as a key gamification element that visualized their progress against the prior year's results and calculated improvement scores. A proprietary algorithm generated scores based on nine parameters: BMI, metabolic syndrome, blood pressure, lipids, uric acid, blood glucose, liver function, urine protein, and visual acuity. As an incentive, the top eight employees demonstrating the most significant improvements or maintenance of health from 2019 to 2020 received rewards and prizes (incentivization). Furthermore, to promote early registration and participation, 100 rewards were distributed by lottery to participants who signed up during the initial phase of the program. Notably, the program did not provide personalized health goals or individual counseling.

The control group consisted of non-participants in the 2020 “Checkup Championship” who underwent health checkups in both 2019 and 2020, with available health checkup data.

### 2.3 Outcome

BMI (kg/m^2^) was selected as the primary outcome of this study for several reasons. First, it is a globally recognized, standardized measure for classifying overweight and obesity (World Health Organization, 2023), and changes in this established health risk indicator are valuable to evaluate. Second, BMI allows comparison with a large body of public health literature, including workplace wellness programs, which commonly report outcomes using BMI or its categories (Asia Pacific Cohort Studies Collaboration, 2006; Flegal et al., 2013; Global BMI Mortality Collaboration et al. 2016; Jayedi et al., 2018; Safaei et al., 2021). BMI is also a simple, non-invasive method widely used in interventions targeting obesity among employees (Anderson et al., 2009; Bezzina et al., 2024) and other populations (Hillier-Brown et al., 2014).

BMI was calculated from height and weight measured during routine annual health checkups. Trained medical staff followed standardized procedures. Body weight (kg) was measured using calibrated medical scales, with participants in light clothing and no shoes. Height (m) was measured with a stadiometer, with participants standing upright without shoes. BMI was calculated as weight divided by height squared. Changes in BMI from 2019 to 2020 were compared between participants and non-participants of the “Checkup Championship.” To confirm robustness and address the use of weight change in other studies, we conducted sensitivity analyses using absolute weight change (kg) as an outcome.

### 2.4 Level of health interest

Health interest was measured in 2020 using the question, “How interested are you in your own health?” Responses were recorded on a 10-point scale (0: not at all interested, 10: very interested). Participants were categorized into three groups based on the tertiles of the health interest score distribution: low health interest (scores below 8), middle health interest (scores of 8 and 9), and high health interest (score of 10).

### 2.5 Covariates

Adjustment variables were extracted from the interview data collected during the 2020 medical examination. Socioeconomic characteristics included sex, age, and household composition. Working condition variables encompassed average monthly working hours and management position. Lifestyle habits, such as smoking, alcohol consumption, exercise habits, and sleep quality, were obtained from the 2019 medical examination to reflect pre-program lifestyle behaviors.

### 2.6 Statistical analysis

Summary statistics for each variable were presented as numbers and percentages (%) stratified by health interest levels. A χ-square test was performed for differences in percentages by health interest groups.

The association between participation in the “Checkup Championship” and BMI changes between 2019 and 2020 was assessed by comparing pre- and post-implementation BMI values. Analyses were conducted using a linear regression model stratified into three groups: low, middle, and high health interest groups. The dependent variable was BMI change from 2019 to 2020, whereas the independent variable was participation in the “Checkup Championship.” Adjustments were made for smoking, alcohol consumption, exercise habits, and sleep quality in 2019, as well as sex, age, household composition, average monthly working hours, and management position in 2020. To examine the interaction effect between program participation and health interest level on BMI, we employed a generalized linear model that included main effects for Time (2019, 2020), Program Participation, and Health Interest Level, as well as their two-way interactions. This model allowed us to estimate BMI at each time point as well as assess differences in BMI trajectories across subgroups.

Inverse probability weighting (IPW) of propensity scores (Austin and Stuart, 2015; Chesnaye et al., 2022) was used to adjust for background factors for participation in the “Checkup Championship.” Propensity scores were derived using logistic regression model. Based on prior literature (Lerman and Shemer, 1996; Nagata et al., 2022) suggesting potential influences on both participation and BMI change, the model included the following pre-participation variables from 2019 and 2020: sex, age, management position, household composition, smoking status, alcohol consumption, quality of sleep, exercise habits, average monthly working hours, health interest score, baseline BMI, baseline waist circumference, baseline blood pressure, baseline LDL cholesterol level, and baseline HbA1c level. We assessed covariate balance before and after IPW by examining standardized differences for all included covariates, with values less than 0.1 indicating adequate balance (Austin and Stuart, 2015) (see eTable 1 for details). The Average Treatment Effect (ATE) of program participation on one-year BMI change was estimated using a weighted linear regression model adjusted for baseline covariates.

To assess the robustness of our findings and enhance comparability with studies focusing specifically on weight change, we conducted a sensitivity analysis using the change in absolute weight (kg) between 2019 and 2020 as an alternative outcome. This sensitivity analysis employed the same IPW-weighted linear regression approach with identical covariate adjustments as the primary BMI analysis.

Missing values were identified primarily in lifestyle covariates (e.g., smoking habits, alcohol consumption, exercise habits, quality of sleep, and average working hours). To address this, multiple imputations were conducted using the fully conditional specification method (*n* = 20). The imputation model included variables with missing data as well as complete variables (such as BMI) as auxiliary to improve imputation accuracy.

All analyses were conducted using R version 4.4.2 (R Foundation for Statistical Computing, Vienna, Austria), with a two-tailed significance level of <0.05.

All individuals involved in this study provided informed consent. This study was approved by the Ethics Committee of Medical Ethics of Kyoto University Graduate School of Medicine and School of Medicine, Kyoto, Japan (approval number: R3057–1).

## 3 Results

Among the 3172 employees, 800 responded to the 2020 health interest question. Of these, 206 (25.8 %) belonged to the low health interest group, 309 (38.6 %) to the middle health interest group, and 285 (35.6 %) to the high health interest group. Table 1 presents the characteristics of participants based on health interest levels. A higher percentage of smokers was observed in the lower health interest group. Individuals without exercise habits also tended to report lower health interest levels. Moreover, participation in the “Checkup Championship” was less frequent among those with lower health interests.

**Table 1:** Characteristics of adult employee participants in Japan (2020) included in the analysis, by health interest level.

	All (800)	Health interest level⁎						*p*-value by χ^2^-test
		Low (206)		Middle (309)		High (285)		
	n	n	%	n	%	N	%	
Sex								
Women	180	52	25.2	67	21.7	61	21.4	0.55
Men	620	154	74.8	242	78.3	224	78.6	
Age								
20's	116	34	16.5	49	15.9	33	11.6	0.09
30's	183	57	27.7	66	21.4	60	21.1	
40's	200	54	26.2	75	24.3	71	24.9	
>50's	301	61	29.6	119	38.5	121	42.5	
Management position								
No	607	163	79.1	235	76.1	209	73.3	0.33
Yes	193	43	20.9	74	24.0	76	26.7	
Household composition								
Single or with siblings or friends	207	65	31.6	75	24.3	67	23.5	0.22
Only two members	192	41	19.9	76	24.6	75	26.3	
Two or more generations	401	100	48.5	158	51.1	143	50.2	
Smoking								
No smoking	430	102	49.5	172	56.0	156	54.9	< 0.01
Quit smoking	129	22	10.7	48	15.6	59	20.8	
Smoking	237	81	39.5	87	28.3	69	24.3	
Alcohol consumption								
Rarely drink	125	34	16.6	49	16.0	42	14.8	0.92
Sometimes drink	375	100	48.8	141	45.9	134	47.2	
Almost daily drink	296	71	34.6	117	38.1	108	38.0	
Exercise habits								
Weekly	345	47	22.9	140	45.6	158	55.6	< 0.01
Monthly	213	64	31.2	84	27.4	65	22.9	
Rarely	238	94	45.9	83	27.0	61	21.5	
Quality of sleep								
Good	422	116	56.6	156	50.8	150	52.8	0.44
Poor	374	89	43.4	151	49.2	75	47.2	
Average working hours per month								
Short	191	42	26.9	64	27.2	85	37.9	0.04
Medium	256	66	42.3	99	42.1	91	40.6	
Long	168	48	30.8	72	30.6	48	21.4	
“Checkup Championship”								
Non-participants	390	122	59.2	140	45.3	128	44.9	< 0.01
Participants	410	84	40.8	169	54.7	157	55.1	

Low: Score < 8, Moderate: Score = 8 or 9, High: Score = 10.

⁎ Health Interest Level: Defined by responses to a 10-point scale (0 = not at all interested, 10 = very interested).

Changes in BMI from 2019 to 2020 are shown in Table 2. Overall, BMI reduction was greater among participants than non-participants (−0.12 vs. -0.36). Furthermore, greater BMI reductions were observed among program participants in the low and middle health interest groups.

**Table 2:** Change in body mass index from 2019 to 2020 among adult employees in Japan, by health interest level⁎ and participation in the “Checkup Championship” program.

	2019	2020	Amount of change from 2019 to 2020
	Mean (SD†)	Mean (SD†)	Mean (SD†)
All subjects			
Non-participants	23.4 (3.6)	23.3 (3.7)	-0.1 (1.0)
Participants	23.6 (3.4)	23.2 (3.2)	−0.4 (1.2)
Low health interest			
Non-participants	23.4 (3.7)	23.4 (3.8)	−0.1 (0.9)
Participants	23.1 (3.3)	22.8 (3.2)	−0.3 (1.0)
Middle health interest			
Non-participants	23.7 (3.8)	23.7 (3.8)	−0.0 (0.9)
Participants	23.5 (3.6)	23.2 (3.4)	−0.3 (1.2)
High health interest			
Non-participants	23.1 (3.4)	22.8 (3.4)	−0.3 (1.0)
Participants	23.9 (3.1)	23.5 (3.0)	−0.4 (1.3)

Low: Score < 8, Moderate: Score = 8 or 9, High: Score = 10.

⁎ Health Interest Level: Defined by responses to a 10-point scale (0 = not at all interested, 10 = very interested).

† SD: Standard deviation.

Table 3 summarizes the estimated effects of “Checkup Championship” on BMI change by health interest level from 2019 to 2020. In the low (estimate: −0.34, 95 % confidence interval [CI]: −0.61, −0.08) and middle (estimate: -0.30, 95 % CI: −0.55, −0.06) health interest groups, BMI reduction was greater in the “Checkup Championship” participation group. No significant BMI reduction was found among participants with high health interests (estimate: -0.16, 95 % CI: −0.44, 0.12). Sensitivity analyses using the change in absolute weight (kg) as the outcome yielded results consistent with these findings for BMI change across all health interest levels (see eTable 2 for details).

**Table 3:** Effects of participation in the “Checkup Championship” program on changes in body mass index from 2019 to 2020 among adult employees in Japan, by health interest level⁎.

	Non-IPW†						IPW†		
	Univariate analysis			Multivariable analysis‡			Multivariable analysis‡		
	Coefficient	95 % CI¶		Coefficient	95 % CI¶		ATE§	95 % CI¶	
All subjects									
Non-participants	1.00			1.00			1.00		
Participants	−0.24	−0.39	−0.09	−0.23	−0.38	−0.08	−0.22	−0.37	−0.07
Low health interest									
Non-participants	1.00			1.00			1.00		
Participants	−0.27	−0.33	−0.21	−0.32	−0.59	−0.04	−0.34	−0.61	−0.08
Middle health interest									
Non-participants	1.00			1.00			1.00		
Participants	−0.27	−0.32	−0.21	−0.31	−0.56	−0.06	−0.30	−0.55	−0.06
High health interest									
Non-participants	1.00			1.00			1.00		
Participants	−0.17	−0.23	−0.11	−0.15	−0.43	0.14	−0.16	−0.44	0.12

⁎ Health Interest Level: Defined by responses to a 10-point scale (0 = not at all interested, 10 = very interested). Low: Score < 8, Moderate: Score = 8 or 9, High: Score = 10.

† IPW: Inverse probability weighting.

‡ Models were adjusted for smoking, alcohol consumption, exercise habits, and quality of sleep in 2019; and sex, age, household composition, average monthly working hours, and management position in 2020.

§ ATE: Average treatment effect.

¶ CI: Confidence interval.

**Fig. 1:**
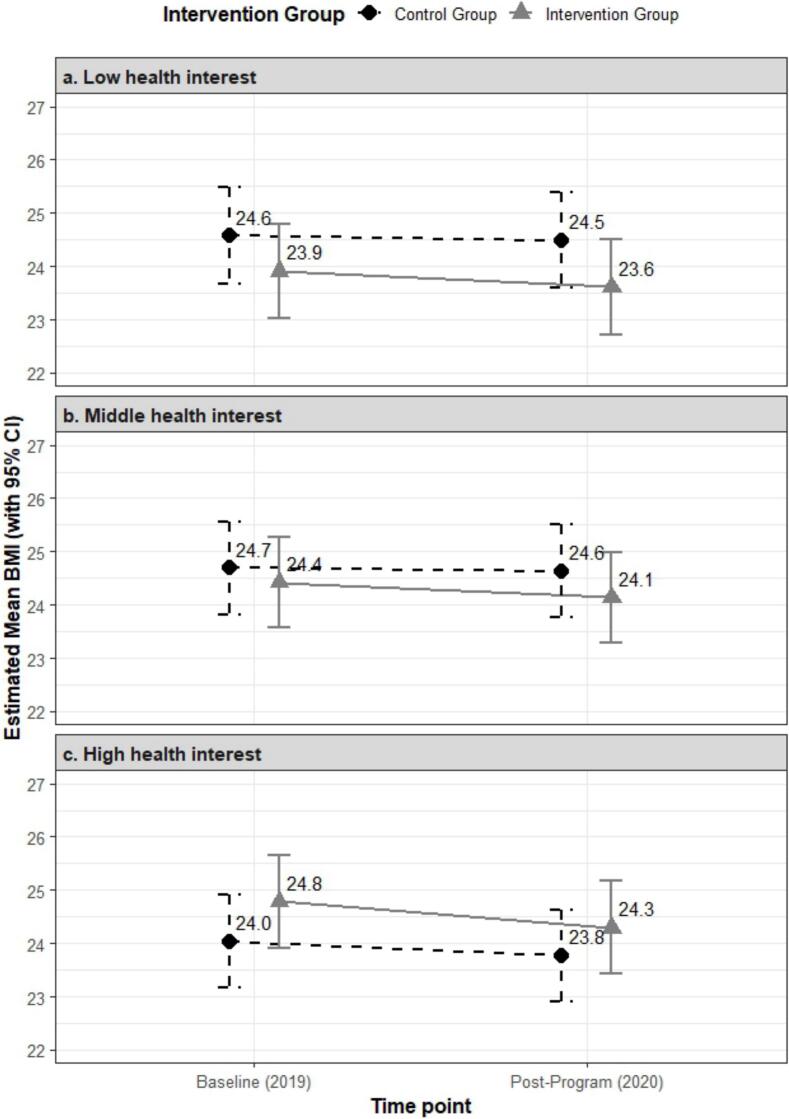
Estimated mean body mass index (BMI) at baseline (2019) and post-intervention (2020) among adult employees in Japan, by health interest level and participation in the “Checkup Championship” program.Estimates were derived from a generalized linear model with BMI at each time point as the dependent variable. The model included main effects for time point (2019 vs. 2020), program participation (yes vs. no), health interest level (low, middle vs. high [reference]), and key two-way interaction terms (Time × Participation, Time × Health Interest, Participation × Health Interest). Models were weighted by inverse probability of program participation and adjusted for smoking, alcohol consumption, exercise habits, and quality of sleep in 2019 and sex, age, household composition, average monthly working hours, and management position in 2020. Error bars represent 95 % confidence intervals (CIs).Health Interest Level:Defined by responses to a 10-point scale (0 = not at all interested, 10 = very interested).Low: Score < 8, Moderate: Score = 8 or 9, High: Score = 10.

Table 4 presents the results from the generalized linear model examining the interaction between program participation and health interest level. Compared to the high health interest group (reference), program participation was associated with significantly lower BMI in both the low (interaction estimate: −1.41, 95 % CI: −2.22, −0.59) and middle (interaction estimate: −1.01, 95 % CI: −1.75, −0.28) health interest groups. Fig. 1 illustrates the estimated marginal mean BMI values in 2019 and 2020 by program participation and health interest level. Program participants exhibited a trajectory of BMI reduction or maintenance across all groups, particularly in the low and middle health interest groups.

**Table 4:** Results from the generalized linear model⁎ examining the interaction between program participation and health interest level† on body mass index among adult employees in Japan.

	Coefficient	95 % CI‡	
Time: Post-Program (2020)	−0.27	−0.88	0.33
“Checkup Championship” participation	0.75	0.14	1.36
Low health interest	0.53	−0.17	1.24
Middle health interest	0.65	0.01	1.29
Post-Program (2020) × “Checkup Championship” participation	−0.22	−0.84	0.40
Post-Program (2020) × Low health interest	0.19	−0.61	0.99
Post-Program (2020) × Middle health interest	0.21	−0.50	0.93
“Checkup Championship” participation × Low health interest	−1.41	−2.22	−0.59
“Checkup Championship” participation × Middle health interest	−1.01	−1.75	−0.28

Low: Score < 8, Moderate: Score = 8 or 9, High: Score = 10.

⁎ The model included main effects for time point (pre-program 2019 vs. post-program 2020), program participation (yes vs. no), health interest level (low, middle vs. high [reference]), and key two-way interaction terms (Time × Participation, Time × Health Interest, Participation × Health Interest). Models were weighted by inverse probability of program participation and adjusted for smoking, alcohol consumption, exercise habits, and quality of sleep in 2019 and sex, age, household composition, average monthly working hours, and management position in 2020.

† Health Interest Level:Defined by responses to a 10-point scale (0 = not at all interested, 10 = very interested).

‡ CI: Confidence interval.

## 4 Discussion

This study examined the effects of a health promotion program incorporating behavioral science on BMI, considering different levels of health interest. Overall, participation in the program contributed to BMI reduction, with a stronger effect observed in the low and middle health interest groups. Although the average reduction in BMI was modest, its clinical relevance should be considered from several perspectives. First, even small changes in BMI at a population level can contribute to a reduction in the prevalence of chronic conditions (Kearns et al., 2014), such as hypertension (Jayedi et al., 2018) and diabetes (Asia Pacific Cohort Studies Collaboration, 2006). Second, this widely accessible and low-intensity program design, which does not include personalized counseling, positions it as a practical approach to primary prevention. In this context, the observed effects, particularly in groups with initially lower health interest, may represent an important first step toward healthier behaviors and potentially greater, sustained improvements over time.

The suppression of BMI increase among participants in the low and middle health interest groups may be attributed to the program's design, which incorporated principles from behavioral science and nudge theory. Several factors, including ease of choice, gamification, commitment, synchronization effect, and timely program implementation (The Behavioural Insights Team, 2024), likely played a role in promoting weight loss behavior. Employees with low health interests often worked long hours, and the simple one-click participation feature may have facilitated engagement among individuals facing time constraints that hinder healthy choices. The program was introduced one and a half months before the scheduled health checkup, aligning with a period of heightened health awareness. Implementing the program within a shorter timeframe may have helped sustain motivation and encouraged behavioral changes. Commitment-driven psychological reinforcement, entertainment elements, and the synchronization effect with social norms—enhanced by the company-wide nature of the program—likely contributed to sustained weight loss efforts. These combined nudging strategies may explain the greater BMI reduction observed among participants compared to non-participants.

However, in the high health interest group, participation in the program had no effect on weight loss. Regardless of participation, this group demonstrated some ability to control BMI increases. Individuals with high health interest likely engaged in healthy behaviors voluntarily, without requiring specific interventions such as the study program. Small stimuli, including program recruitment efforts and the workplace environment associated with the “Checkup Championship,” may have been sufficient to promote their health improvements.

The study also highlighted key characteristics of individuals with low health interests. A greater likelihood of working long hours and negative associations with health behaviors—such as smoking, lack of exercise, and non-participation in the program—were observed in this group. Although the classification of health interest was relative, negative associations with health behaviors aligned with findings from previous studies (Iversen and Kraft, 2006; Nishizawa et al., 2024; Pu et al., 2020). These findings suggest that individuals with low health interests should be prioritized as the main target for the “Checkup Championship.” As the majority of the study population exhibited high health interest levels (705 respondents, 88.1 % scored 7 or higher), a relative classification was applied to assess the program's effects. Therefore, stronger negative associations with health behaviors may emerge in individuals with even lower absolute health interests. Recently, the interest in health scale has been developed for the Japanese population (Ozawa C, Ishikawa H, Kato M, Fukuda Y, 2021), with a proposed cutoff value (Nishizawa et al., 2024). Applying this scale could facilitate the identification of individuals with absolute health indifference. To better understand the characteristics of this population and evaluate program effectiveness, modifications to the “Checkup Championship” and new strategies for encouraging participation should be considered.

This study suggests that a nudge-applied health promotion program is an effective intervention for the health-indifferent population and helps reduce health disparities. Several studies on the effects of nudge-based health programs on health disparities have been reported in countries other than Japan (Harbers et al., 2020; Schüz et al., 2021). In Japan, nudge-based health promotion programs have also been implemented (Nagatomo et al., 2019; Takebayashi et al., 2022, Takebayashi et al., 2024), although their impact on health disparities has been limited. For example, financial incentives may increase vegetable intake more effectively among non-fulltime workers with lower incomes (Nagatomo et al., 2019). Evaluating the effectiveness of health promotion programs on health disparities is challenging. However, examining the effects of various programs, with or without nudge theories, on health disparities while understanding the characteristics of the target population, such as health interests and SES, is essential, as demonstrated in this study.

This study had some limitations. First, the generalizability of the results was limited because the study was conducted within a single firm, and the number and demographics of the participants were restricted. Second, the non-randomized controlled trial design introduced a risk of selection bias and made it difficult to eliminate the influence of unmeasured confounders when estimating causal relationships. However, efforts were made to reduce this limitation by simulating a quasi-randomized controlled trial using IPW with propensity scores to align background factors as closely as possible. Despite these efforts using measured covariates, the potential influence of unmeasured confounders cannot be fully eliminated. As a result, it remains inherently difficult to determine whether the observed differences are attributable to participants' health interest or to other measured and unmeasured factors. Furthermore, although the “Checkup Championship” began in 2019, the analysis did not adjust for participation status in 2019, which may have led to an underestimation of the actual impact. The effects of continuous participation were not fully explored, highlighting the need for further analyses that consider changes in health interest over time. Future research should examine the long-term impact of sustained participation to provide a more comprehensive evaluation. Third, the single-item question used to assess health interest may have led to variations in how respondents interpreted “health” and “interest,” potentially influencing the results. Furthermore, this measure has not been formally evaluated for its validity or reliability, adding further uncertainty to the findings related to health interest. Future research would benefit from using established multi-item scales with demonstrated psychometric properties, such as the interest in health scale (Ozawa et al., 2021), to more reliably assess this construct. Additionally, the 2020 intervention occurred during the COVID-19 pandemic. The shift to remote work and other pandemic-related societal changes may have independently influenced employees' lifestyle and health outcomes. Finally, due to insufficient data on lifestyle and environmental factors, we could not determine the specific changes that contributed to successful weight loss from participation in the “Checkup Championship.”

## 5 Conclusion

The “Checkup Championship” program, designed based on behavioral science and nudge theory, contributed to BMI reduction in the middle and low health interest groups. This study suggests that the program may be effective for improving BMI in populations with these health interest levels, which are considered to have a high need for health promotion interventions. Based on this study results, future research is necessary to examine the long-term impact of sustained participation while clarifying the absolute health indifference group.

## CRediT authorship contribution statement

**Takuya Yamada:** Writing – review & editing, Writing – original draft, Visualization, Software, Methodology, Investigation, Formal analysis, Data curation, Conceptualization. **Kumi Sugimoto:** Writing – original draft, Software, Methodology, Investigation, Formal analysis, Data curation, Conceptualization. **Hanae Nagata:** Writing – review & editing, Validation, Software, Project administration, Methodology, Investigation, Formal analysis. **Yoshiharu Fukuda:** Writing – review & editing, Supervision, Methodology, Conceptualization. **Koryu Sato:** Writing – review & editing, Validation, Software, Methodology, Formal analysis. **Naoki Kondo:** Writing – review & editing, Supervision, Resources, Project administration, Methodology, Funding acquisition, Conceptualization.

All authors have reviewed the final version of the manuscript and approved it for submission.

## Funding

This work was supported by JSPS KAKENHI Grant Numbers 18H04071 and 19H01076; Grants-in-Aid for Scientific Research from the Japanese Ministry of Health, Labour and Welfare (Grant Numbers 19FA1011 and 22FA1001); Hakuhodo DY Holdings Inc. and Hakuhodo DY Media Partners Inc.

## Declaration of competing interest

The authors declare the following financial interests/personal relationships which may be considered as potential competing interests: Naoki Kondo received research funding from Hakuhodo DY Holdings Inc. and Hakuhodo DY Media Partners Inc. The funds were allocated for conducting epidemiologic studies using the data provided by the company. The funding agreement imposed conditions on data availability (e.g., prohibiting the publication of data related to the company's salary structure) but did not impose any restrictions on selecting study topics, study designs, analysis, interpretation, writing of the article, or submission decisions. Additionally, NK advised the company on evaluating the studied program after its implementation and had no role in designing the program.

## Data Availability

The authors do not have permission to share data.

## Appendix A Supplementary data

Supplementary material Image 1
