# Supplementary material for: Heterogeneous effects on body mass index in the “checkup championship”: A behavioral science-based health promotion program by health interest level
Source: Prev Med Rep. 2025 Jun 28;56:103153. doi: 10.1016/j.pmedr.2025.103153 (PMC12272467; doi:10.1016/j.pmedr.2025.103153)
Supplement: Supplementary file 1 — Supplementary material [file mmc1.docx]

**Online Supplementary Material**

**Heterogeneous effects of weight loss in the “Checkup Championship”: A nudge-based health promotion program by health interest level**

**eTable 1.** Comparison of the means between adult employee participants and non-participants in Japan before and after weighting.

|  | Before weighting | | |  | After weighting | | |
| --- | --- | --- | --- | --- | --- | --- | --- |
|  | Participant | Non-  participant | Standardized  difference |  | Participant | Non-  participant | Standardized  difference |
| Sex: Women | 20.5 | 24.6 | 0.1 |  | 22.7 | 23.2 | 0.0 |
| Age: ≥ 40’s | 63.9 | 61.3 | 0.1 |  | 62.4 | 62.2 | 0.0 |
| Management position: No | 72.2 | 79.7 | 0.2 |  | 75.6 | 76.2 | 0.0 |
| Household composition: Single or with siblings or friends | 22.4 | 29.5 | 0.2 |  | 26.1 | 26.1 | 0.0 |
| Smoking | 26.0 | 33.9 | 0.2 |  | 29.7 | 30.1 | 0.0 |
| Alcohol consumption: Almost daily drink | 40.0 | 34.3 | 0.1 |  | 37.1 | 37.4 | 0.0 |
| Exercise habits: Rarely | 24.2 | 35.9 | 0.3 |  | 29.8 | 30.3 | 0.0 |
| Quality of sleep: Poor | 47.5 | 46.4 | 0.0 |  | 47.0 | 47.0 | 0.0 |
| Average working hours per month: Long | 29.9 | 26.9 | 0.2 |  | 28.5 | 28.2 | 0.0 |
| Level of Health interest: Low | 20.5 | 31.2 | 0.3 |  | 24.8 | 26.9 | 0.2 |
| Body mass index in 2019 | 23.6 | 23.4 | 0.0 |  | 23.4 | 23.4 | 0.0 |
| Systolic blood pressure in 2019 | 118.4 | 118.5 | 0.0 |  | 118.3 | 118.3 | 0.0 |
| Diastolic blood pressure in 2019 | 73.2 | 73.7 | 0.0 |  | 73.4 | 73.4 | 0.0 |
| Low-density lipoprotein cholesterol in 2019 | 121.3 | 120.7 | 0.0 |  | 120.9 | 120.9 | 0.0 |
| Hemoglobin A1c in 2019 | 5.2 | 5.2 | 0.1 |  | 5.2 | 5.2 | 0.0 |

**eTable 2.** Effects of participation in the “Checkup Championship” program on changes in weight (kg) from 2019 to 2020 among adult employees in Japan, by health interest level^*^.

|  | Non-IPW^†^ | | | | | | |  | IPW^†^ | | |
| --- | --- | --- | --- | --- | --- | --- | --- | --- | --- | --- | --- |
|  | Univariate analysis | | |  | Multivariable analysis^‡^ | | |  | Multivariable analysis^‡^ | | |
|  | Coefficient | 95% CI^¶^ | |  | Coefficient | 95% CI^¶^ | |  | ATE^§^ | 95% CI^¶^ | |
| All subjects |  |  |  |  |  |  |  |  |  |  |  |
| Non-participants | 1.00 |  |  |  | 1.00 |  |  |  | 1.00 |  |  |
| Participants | -0.71 | -1.16 | -0.27 |  | -0.68 | -1.14 | -0.24 |  | -0.64 | -1.08 | -0.20 |
| Low health interest |  |  |  |  |  |  |  |  |  |  |  |
| Non-participants | 1.00 |  |  |  | 1.00 |  |  |  | 1.00 |  |  |
| Participants | -0.80 | -1.57 | -0.03 |  | -0.92 | -1.73 | -0.12 |  | -0.97 | -1.74 | -0.20 |
| Middle health interest |  |  |  |  |  |  |  |  |  |  |  |
| Non-participants | 1.00 |  |  |  | 1.00 |  |  |  | 1.00 |  |  |
| Participants | -0.81 | -1.52 | -0.10 |  | -0.97 | -1.70 | -0.23 |  | -0.94 | -1.66 | -0.23 |
| High health interest |  |  |  |  |  |  |  |  |  |  |  |
| Non-participants | 1.00 |  |  |  | 1.00 |  |  |  | 1.00 |  |  |
| Participants | -0.47 | -1.29 | 0.34 |  | -0.40 | -1.25 | 0.45 |  | -0.41 | -1.23 | 0.41 |

^*^Health Interest Level:

Defined by responses to a 10-point scale (0 = not at all interested, 10 = very interested). Low: Score < 8, Moderate: Score = 8 or 9, High: Score = 10.

^†^IPW: Inverse probability weighting.

^‡^Models were adjusted for smoking, alcohol consumption, exercise habits, and quality of sleep in 2019; and sex, age, household composition, average monthly working hours, and management position in 2020.

^§^ATE: Average treatment effect.

^¶^CI: Confidence interval.
